# Genetically predicted hypertension, antihypertensive drugs, and risk of erectile dysfunction: a Mendelian randomization study

**DOI:** 10.3389/fcvm.2023.1157467

**Published:** 2023-06-09

**Authors:** Cong Zhao, Jun-long Feng, Sheng Deng, Xiang-peng Wang, Yu-jie Fu, Bin Wang, Hai-song Li, Fan-chao Meng, Ji-sheng Wang, Xian Wang

**Affiliations:** ^1^ Department of Andrology, Dongzhimen Hospital, Beijing University of Chinese Medicine, Beijing, China; ^2^ Department of Andrology, Shunyi Hospital, Beijing Hospital of Traditional Chinese Medicine, Beijing, China; ^3^ Graduate School, Beijing University of Chinese Medicine, Beijing, China; ^4^ Urology Surgery, The Third Affiliated Hospital of Beijing University of Chinese Medicine, Beijing, China; ^5^ Department of Cardiology, Dongzhimen Hospital, Beijing University of Chinese Medicine, Beijing, China

**Keywords:** hypertension, antihypertensive drugs, erectile dysfunction, Mendelian randomization study, blood pressure

## Abstract

**Background:**

The causal relationship between hypertension, antihypertensive drugs and the risk of erectile dysfunction is still uncertain. We performed a univariable and multivariable Mendelian randomization study to investigate whether they are causally related to erectile dysfunction.

**Methods:**

Genetic variants associated with blood pressure were derived from the genome-wide association study meta-analysis of the UK Biobank and International Consortium of Blood Pressure (*N* = 757,601). Summary association data for hypertension were obtained from the UK Biobank (*N* = 463,010) and the FinnGen study (*N* = 356,077). The summary statistics of erectile dysfunction were obtained from the European ancestry with 223,805 subjects. The SNP instruments used to assess the effect of the protein targets of antihypertensive drugs on erectile dysfunction were obtained from previous studys. Causal effects were estimated using the univariate Mendelian randomization method (inverse variance weighted, MR-Egger, weighted median, MR-PRESSO and Wald ratios) and the multivariate Mendelian randomization method. Sensitivity analyses were implemented with the Cochran's *Q*-test, MR-Egger intercept test, MR-PRESSO, and leave-one-out analysis.

**Results:**

Univariate MR found that elevated diastolic blood pressure may increase the occurrence of erectile dysfunction (odds ratio [OR] = 1.012; 95% confidence interval [CI]: 1.000–1.024; *P *= 0.047). Genetically predicted hypertension is also associated with ED (For the FinnGen, OR = 1.106; 95% CI: 1.027–1.191; *P* = 0.008. For the UK Biobank, OR = 3.832; 95% CI: 1.410–10.414; *P *= 0.008). However, after adjusting for systolic blood pressure, diastolic blood pressure and hypertension using multivariate Mendelian randomization, only hypertension was causally associated with ED occurrence (For the FinnGen, OR = 1.103; 95% CI: 1.018–1.195; *P *= 0.017. For the UK Biobank, OR = 5.037; 95% CI: 1.601–15.846; *P *= 0.006). We found no evidence that the use of angiotensin-converting enzyme inhibitors, beta-blockers, calcium channel blockers, and thiazide diuretic increased the risk of erectile dysfunction.

**Conclusions:**

Genetically predicted hypertension increases the risk of erectile dysfunction, but we found no causal relationship between elevated systolic/diastolic blood pressure and erectile dysfunction. We speculate that the relationship between elevated blood pressure and erectile dysfunction risk may be nonlinear. We found little evidence that antihypertensive drugs increase the risk of erectile dysfunction.

## Introduction

Erectile dysfunction (ED) is common in adult men. The European Male Aging Study (EMAS) reports a prevalence of ED ranging from 6% to 64% depending on the age subgroup and increasing with age (1). The erectile process involves complex psychological factors, sex hormone levels, and alterations at the neurovascular level (2). ED is mainly classified as psychological and organic. Organic factors mainly involve lesions in vascular endothelial function and share many risk factors with hypertension. Indeed, hypertension is a risk factor for ED (3), with more than 40% of men with ED also diagnosed with hypertension, demonstrating that ED may be an early sign of hypertension (4).

There is a need to investigate whether antihypertensive drugs affect ED since hypertension may be a risk factor for ED and antihypertensive drugs are the main treatment for hypertension. In addition, some patients resist treatment because of concerns about the adverse effects of antihypertensive drugs on sexual function. The European Society of Hypertension (ESH) and European Society of Cardiology (ESC) guidelines for the management of arterial hypertension state that thiazide diuretics and beta-adrenoceptor blockers (BBs) may induce or exacerbate male sexual dysfunction, whereas angiotensin-converting enzyme inhibitors (ACEIs) and calcium channel blockers (CCBs) may have neutral or even beneficial effects on erectile function (5). However, most of these recommendations come from systematic evaluations of observational or interventional studies and expert opinions. Randomized controlled trials (RCT) studies are rare and have generated conflicting conclusions (6, 7).

Traditional RCTs have some drawbacks. ED patients are mostly associated with endocrine comorbidities, making it more difficult to observe the causal relationship between hypertension and ED. In addition, socio-cultural barriers reduce the reporting rate of ED. Moreover, previous studies have used different questionnaires to assess erectile function, making clinical trials for ED somewhat heterogeneous. Finally, patients' perceptions of drugs on potential adverse events bias can lead to the Hawthorne effect, which can further exacerbate sexual dysfunction (8) and exaggerate the incidence of drug side effects (9).

Mendelian randomization (MR) is an emerging form of instrumental variable analysis that uses genetic variants randomly assigned to loci at the time of conception as instrumental variables to mimic randomization in randomized controlled trials (10, 11). Where individual-level data are unavailable, pooled data on single nucleotide polymorphisms (SNPs) from separate genome-wide association studies (GWAS) can be used for two-sample MR to investigate the association and causality of exposure and outcome. This study determined whether systolic blood pressure (SBP), diastolic blood pressure (DBP) and hypertension are causally associated with ED. In addition, it analyzed the possible effect of common antihypertensive drugs on ED by examining genetic variants in their protein target genes.

## Methods

### Study design

**Figure 1:**
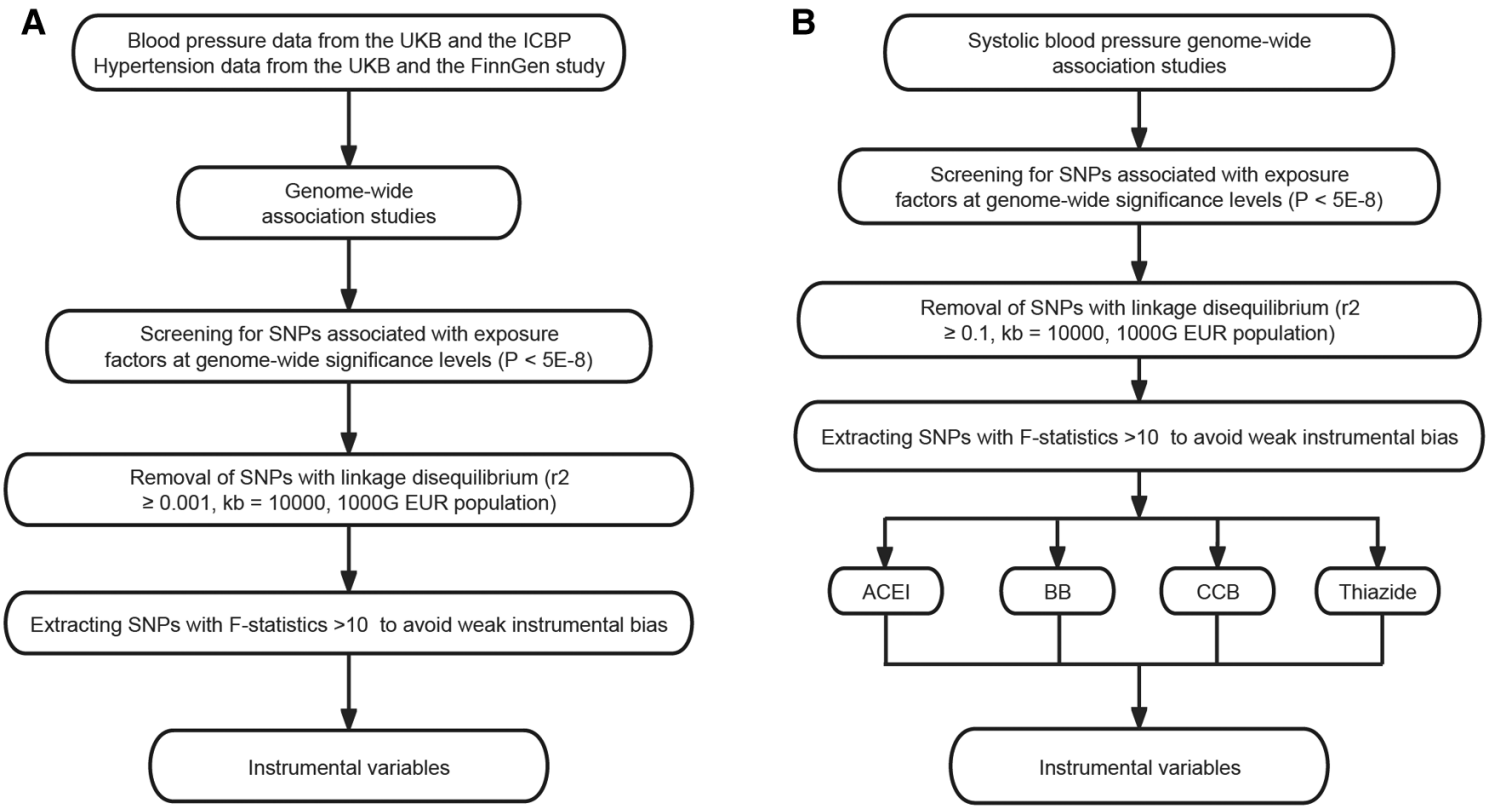
Selection of instrumental variables. (**A**) Selection of instrumental variables for blood pressure and hypertension. (**B**) Selection of instrumental variables for antihypertensive drug acting protein targets. ICBP, International Consortium of Blood Pressure; SNP, single-nucleotide polymorphism; ACEI, angiotensin-converting enzyme inhibitor; BB, beta-adrenoceptor blocker; CCB, calcium channel blocker.

Genetic associations were obtained from pooled data from different GWAS studies (Table 1). First, we performed a two-sample MR to analyze the causal relationship between SBP, DBP, hypertension and ED, followed by the use of published genetic variants in genes regulating drug target proteins (12, 13) as different classes of antihypertensive drug alternatives. Then we assessed the association of these genetic variants with ED in GWAS (Figure 1). All data used were publicly available and were from populations of European ancestry.

**Table 1:** Summary of genome-wide association studies included in this study.

Phenotype	GWAS data source	Cohort(s)	Unit	Sample size	Race
Systolic blood pressure	Evangelou et al., 2018	UK Biobank and ICBP	SD 10 mmHg	757,601	European
Diastolic blood pressure	Evangelou et al., 2018	UK Biobank and ICBP	SD 10 mmHg	757,601	European
Hypertension	UK Biobank	UK Biobank	NA	463,010	European
Hypertension	FinnGen	FinnGen	NA	356,077	European
Erectile dysfunction	Bovijn et al., 2018	UK Biobank, EGCUT, PHB	NA	223,805	European

ICBP, International Consortium of Blood Pressure; EGCUT, the Estonian Genome Center of the University of Tartu; PHB, hospital-recruited Partners HealthCare Biobank.

### Study population

The GWAS dataset of SBP and DBP was obtained from the meta-analysis of 757,601 individuals of European descent in the UK Biobank and International Consortium of Blood Pressure-Genome Wide Association Studies (ICBP) (14).

Summary association data for hypertension were obtained from the UK Biobank (*N* = 463,010, 54,358 cases and 408,652 controls) and the FinnGen study (15) (*N* = 356,077, 101,652 cases and 254,425 controls). Hypertension was defined based on hospital diagnosis, death, and insurance records as patients with SBP consistently ≥140 mmHg or DBP consistently ≥90 mmHg.

The ED GWAS dataset is the largest ED dataset of European ancestry. A total of 223,805 subjects (6,175 cases and 217,630 controls) were recruited by combining three cohorts (16). The diagnosis of ED was based on the International Classification of Diseases, 10th Revision (ICD-10) codes (N48.4 and F52.2), or a history of medical intervention for ED such as surgery or oral medication, or self-reported by the participants. Detailed information is available in the original paper.

This genetic association study used only published abstract data from studies involving human participants, and ethical approval and patient consent were obtained in the original study for which data were used in this study. Therefore, these approvals and patient consent were not required for this study. This study followed the Strengthening the Reporting of Observational Studies in Epidemiology-Mendelian Randomization (STROBE-MR) reporting guidelines (17).

### Instrumental variable selection

MR uses genetic variation as instrumental variables that satisfy the following core assumptions: (1) Relevance: genetic variation must be related to the exposure factor. (2) Independence: The genetic variation must be independent of confounding factors that may affect the exposure-outcome association. (3) Exclusivity: The genetic variation must affect the outcome only through its effect on exposure and not by any other route. These assumptions ensure that genetic variation can be used as a valid tool to estimate the causal effects of exposure on outcomes.

We selected single nucleotide polymorphisms (SNPs) associated with SBP, DBP and hypertension, screening for SNPs with genome-wide significance levels using (*P *< 5 × 10^−8^). When multiple SNPs were used as instruments, aggregation was performed using linkage disequilibrium (LD) between them to identify nearly independent SNPs. LD was calculated based on a European reference group of 1,000 Genomics European reference panels. Single nucleotides with LD were trimmed to single nucleotides with *r^2^* ≥ 0.001 and a window size of 10,000 Kb.

SNPs in ACE, ADRB1, CACN1C, CACN1D, CACNB2, CACNB3, and SLC12A3 (*N*CC) associated with SBP in GWAS were used to proxy inhibition of angiotensin-converting enzyme (ACE), *β*−1 adrenergic receptor (ADRB1), calcium channel blockers (CACN1C, CACN1D, CACNB2, CACNB3) and thiazide diuretics (NCC). For each drug target, SNPs in the drug target gene region (±10 kB ∼ ±100 kB) that were associated with SBP (*P* < 5 × 10^−8^) were selected as proxies for drug target perturbation after clumping to a pairwise linkage disequilibrium threshold of *r*^2^ < 0.1 using the 1,000 Genomes European reference panel.

Avoidance of bias for weak instrumental variables by estimating *F*-statistics to assess instrument strength. The *F*-statistic for all extracted SNPs was >10, indicating no weak instrumental bias. The above steps ensure that the relevance assumptions are met.

### Mendelian randomisation analysis

**Figure 2:**
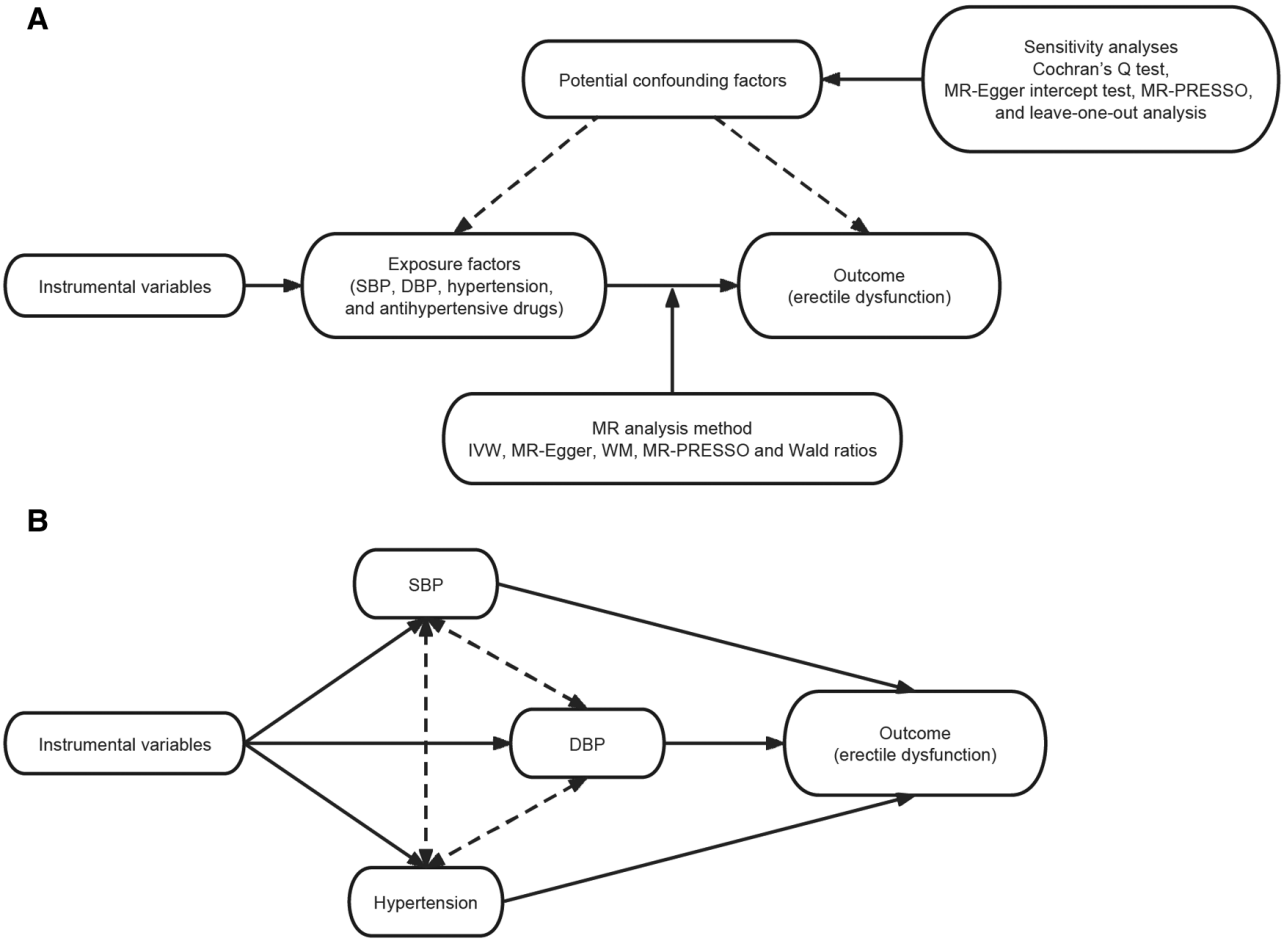
Study flow graph. (**A**) Schematic diagram of the principle of Mendelian randomization and the study process. (**B**) Schematic diagram of the principle of multivariable Mendelian randomization and the study process. SBP, systolic blood pressure; DBP, diastolic blood pressure; IVW, inverse variance weighted; WM, weighted median.

MR analyses were conducted in this study to assess the causal effects of individual exposure factors on ED. We applied the inverse variance weighted (IVW) estimates to the main analyses. In addition, other MR analyses, specifically the MR-Egger and weighted median, complemented the IVW, and these methods provided more reliable estimates across a wider range of scenarios, ensuring robustness of the study results. We performed Wald ratio method where only a single SNP was available (Figure 2).

In addition, considering the existence of common SNPs for SBP, DBP, and hypertension, we performed multivariate MR analysis by MVMR-Lasso method (18) to determine their causal relationship with ED.

### Sensitivity analysis

Mendelian randomization estimates may be influenced by multiplicity and heterogeneity, whereby a selection of proxy-exposed SNPs affects the results through mechanisms different from those expected. We used the MR-Egger intercept (19) to detect directional pleiotropy, the MR-PRESSO method to examine horizontal pleiotropy (20). These tests verify the independence and exclusivity assumption. We used the Cochran's *Q*-test to estimate heterogeneity between genetic instruments. To ensure that causality in the MR analysis was not driven by a single instrumental variable, we performed a leave-one-out analysis, which sequentially removed each SNP (21). Meanwhile, our study was validated in two independent hypertension GWAS databases to ensure the robustness of the results.

### Statistical methods

To account for multiple testing in our analyses, a Bonferroni-corrected threshold of *P* < 0.0042 (*α* = 0.05/12 exposure factors) was applied. Associations with *P* < 0.0046 were considered significant, and associations with *P *≥ 0.0042 and <0.05 were considered suggestive. As we used dichotomous variables for both exposure and outcome, MR estimates were expressed as dominance ratios (ORs) and corresponding 95% confidence intervals (CIs), which provide estimates of the increased risk of outcome caused by each standard deviation (SD) increase for different exposure factors. All analyses were performed by the packages TwoSampleMR (version 0.5.6) and MRPRESSO (1.0) in R (version 4.2.1).

## Results

### Instrument variable selection results

By performing a series of instrumental variable selection steps, we identified 444 SNPs as instrumental variables for SBP, 446 SNPs as instrumental variables for DBP, 154 SNP as instrumental variables for hypertension (FinnGen), 67 SNP as instrumental variables for hypertension (UKB) and 38 SNPs for antihypertensive drugs, corresponding to 8 protein targets for 4 different drugs, respectively (Supplementary Tables S2–S4).

### Blood pressure, hypertension and ED

MR analysis showed that elevation of DBP was associated with ED, whereas SBP had no effect on ED (per 10 mmHg increase in SBP: IVW, OR = 1.006, 95% CI: 0.999–1.013, *P *= 0.107; per 10 mmHg increase in DBP: IVW, OR = 1.012, 95% CI: 1.000–1.024, *P *= 0.047). IVW analysis showed that each standard deviation increase in hypertension increased the risk of ED (For the FinnGen, OR = 1.106; 95% CI: 1.027–1.191; *P* = 0.008. For the UK Biobank, OR = 3.832; 95% CI: 1.410–10.414; *P *= 0.008) (Table 2). None of these results reached the threshold for significance.

**Table 2:** Genetic prediction of the causal relationship between blood pressure, hypertension and erectile dysfunction.

Exposure	N. SNPs	Method	OR (95%CI)	*P*
SBP	444	IVW	1.006 (0.999–1.013)	0.107
DBP	446	IVW	1.011 (1.000–1.024)	0.047
Hypertension (FinnGen)	154	IVW	1.106 (1.027–1.191)	0.008
Hypertension (FinnGen)	136	MVMR-Lasso	1.103 (1.018–1.195)	0.017
Hypertension (UKB)	67	IVW	3.832 (1.410–10.414)	0.008
Hypertension (UKB)	39	MVMR-Lasso	5.037 (1.601–15.846)	0.006

SBP, systolic blood pressure; DBP, diastolic blood pressure; N. SNPs, the number of single nucleotide polymorphism; OR, odds ratio; CI, confidence interval; IVW, inverse variance weighted; MVMR, multivariate mendelian randomization; UKB, UK Biobank.

After adjusting for systolic blood pressure, diastolic blood pressure and hypertension using multivariate Mendelian randomization, only hypertension was causally associated with ED occurrence (For the FinnGen, OR = 1.103; 95% CI: 1.018–1.195; *P *= 0.017. For the UK Biobank, OR = 5.037; 95% CI: 1.601–15.846; *P *= 0.006) (Table 2).

### Antihypertensive drugs and ED

MR analysis found that SBP-lowering drugs (ACEIs, BBs, CCBs and thiazide diuretics) did not affect the risk of ED (Table 3).

**Table 3:** The associations of genetically predicted drugs target with erectile dysfunction.

Drug	Target	N. SNPs	Method	OR (95% CI)	*P*
ACEIs	ACE	12	IVW	1.016 (0.972–1.062)	0.469
BBs	ADRB1	2	IVW	0.949 (0.871–1.034)	0.234
CCBs	All	23	IVW	0.969 (0.939–1.000)	0.051
	CACNA1C	2	IVW	1.036 (0.896–1.197)	0.632
	CACNA1D	4	IVW	0.930 (0.834–1.036)	0.187
	CACNB2	16	IVW	0.976 (0.941–1.012)	0.197
	CACNB3	1	Wald ratio	0.912 (0.791–1.051)	0.202
Thiazide diuretics	NCC	1	Wald ratio	0.915 (0.711–1.179)	0.493

N. SNPs, the number of single nucleotide polymorphism; OR, odds ratio; CI, confidence interval; ACEIs, angiotensin-converting enzyme inhibitors; BBs, beta-adrenoceptor blockers; CCB, calcium channel blocker.

OR equivalent to 1 mm Hg lower systolic blood pressure.

### Sensitivity analysis

Cochran's *Q*-test found no heterogeneity in those outcomes. MR-Egger test was used to identify pleiotropy, and the intercept was observed to be close to zero, indicating no directional pleiotropy. MR-PRESSO did not find horizontal pleiotropy in these results (Supplementary Tables S6, S7). Leave-one-out analysis found no substantial difference in causality even when each SNP was removed in turn. It suggests that no single SNP drives a causal association between exposure factors and outcome (Supplementary Tables S9–S13).

When MR analysis was performed using the UKBB and FinnGen datasets separately, the results were different, which we believe may be related to the winner's bias caused by the sample overlap rate. There was sample overlap between UKBB and the ED dataset, approximately 43% (199,352/ 463,010), whereas there was no sample overlap between FinnGen and the ED dataset. In Mendelian randomization studies, winner's curse bias occurs when there is overlap between the data set for selecting genetic variations and the data set for estimating genetic associations. Jiang et al. (22) found that the winner's curse in outcome association estimates often overstates the estimates of MR, but this bias does not materially affect the results. However, even considering that the OR for causality between UKBB and ED may be exaggerated, we still get a consistent direction of causality in the FinnGen dataset, which verifies the reliability of our conclusions.

## Discussion

In this study, we assessed the causal relationships between hypertension, antihypertensive medications and ED. Our study found no causal relationship between elevated SBP/DBP and ED, but hypertension increased the risk of ED. In addition, we used genetic proxies to assess the effect of antihypertensive medications on ED. We did not find a possible effect of antihypertensive drugs on ED risk.

Physiologically, the hypothesis that hypertension causes ED is possible, because hypertension will lead to a continuous increase in the release of vasoconstrictors (e.g., Angiotensin receptor II, Endothelin-1 and aldosterone). These substances can cause endothelial dysfunction, which adversely affects the erection process (23). Many clinical studies have also analyzed the relationship between hypertension and ED. Michael Doumas et al. (24) found that men with high blood pressure had a higher prevalence of erectile dysfunction than those with normal blood pressure after surveying 634 men. Capri G Foy et al. analyzed 1,255 men in a SBP Intervention Trial (SPRINT) and found that lower SBP and higher DBP were associated with better erectile function in older men with hypertension (25). Wayland Hsiao et al. found in a retrospective cohort study of 39,320 men with hypertension that tighter blood pressure control was associated with a lower incidence of ED and a longer time to ED development (26). In addition, hypertension often coexists with other cardiac metabolic factors (such as hyperglycemia, hyperlipidemia, etc.) Their interaction may aggravate erectile dysfunction, but there is little research on this at present (27). Sarma Aruna V et al. found that higher SBP increased the risk of ED, while antihypertensive drugs could reduce ED occurrence in a prospective cohort study involving 692 type 1 diabetic male patients. These studies all indicate that hypertension has a negative impact on erectile function, but the existing studies have small sample sizes and cannot exclude all confounding factors (such as sex hormone levels, etc.). Considering that hypertension may not have obvious symptoms in the early stage, some researchers believe that ED can be an early manifestation of hypertension and other cardiac diseases (23). But this also brings difficulties to the research, because finding hypertension in ED patients does not prove that hypertension directly causes ED.

Our study used genetically predicted instrumental variables to demonstrate that hypertension does increase ED risk. However, SBP or DBP was not associated with ED risk, demonstrating that the relationship between elevated BP and ED is likely to be nonlinear. Ioakeimidis et al. (28) found that men with high normal blood pressure (130–139/85–89 mmHg) had significant microvascular and macrovascular injury, and that functional changes in the penile vasculature were similar to those in men with stage I arterial hypertension (140–159/90–99 mmHg). This suggests that when blood pressure is within a certain range, its increase does not necessarily increase the risk of ED. Considering that the onset of ED symptoms precedes the detection of hypertension or other cardiovascular disease, we hypothesized that prehypertension may also contribute to ED. Future researchers need to further explore the blood pressure threshold leading to erectile dysfunction, which may contribute to our understanding of novel mechanisms by which hypertension leads to cardiovascular disease.

When MR analysis was performed using the UKBB and FinnGen datasets separately, the results were different, which we believe may be related to the winner curse bias caused by the sample overlap rate. There was sample overlap between UKBB and the ED dataset, approximately 43% (199,352/463,010), whereas there was no sample overlap between FinnGen and the ED dataset.

There is a lack of consensus on the effects of antihypertensive drugs on ED. The European Society of Hypertension (ESH) and European Society of Cardiology (ESC) guidelines for the management of hypertension state that thiazide diuretics and *β*-blockers may induce or exacerbate ED, whereas ACEIs, ARBs, CCBs, and BBs may have a neutral or even beneficial effect on ED (5). However, most of these recommendations are derived from systematic evaluations of observational or interventional studies and expert opinions (29, 30). In a network meta-analysis including 25 studies (7,784 patients) and 16 quantitative pooled studies, Ioannis T. Farmakis et al. found insufficient evidence of a harmful or beneficial effect of any major antihypertensive drug compared with placebo, and the risk of bias and inconsistency was high in most studies (6). Patients' knowledge of the medication and its side effects on antihypertensive drugs may increase the incidence of ED after taking the medication (Hawthorn effect) (31), which may help explain some of the inconsistent findings in RCTs. We analyzed most antihypertensive drugs and specific protein targets of action in this study and found no evidence for an effect of antihypertensive drugs on ED. MR uses genes as instrumental variables, avoiding important influences on ED (e.g., body mass index, diabetes, and smoking) interfering with clinical trial results, making the findings more reliable.

The main advantage of this study is the MR design, which avoids the interference of common influencing factors of ED in clinical trials and reduces the potential bias from confounding factors, making it possible to conduct clinical trials that would not normally be possible. The large sample of summary statistics obtained from GWAS is much larger than the number of patients that can be included in a normal RCT, improving the ability to test for causal effects and making the results more reliable.

This study had several limitations. First, we analyzed the life-long hypotensive effect of antihypertensive drugs on ED using MR, which may differ from the short-term effect of drug treatment. Second, due to the lack of available instrumental variables, only one SNP was obtained for certain drugs or protein targets, which may not provide sufficient statistical efficacy. When using the potential genetic targets of antihypertensive treatment as substitutes for their pharmacological effects, we cannot fully simulate the effects of drugs, such as the mechanisms of action of dihydropyridine and non-dihydropyridine CCBs are not completely the same. The effect of combined antihypertensive regimens on erectile function cannot be explained by a single target. These factors weaken the reliability of the study. Third, the factors that affect ED are very complex. We have only identified hypertension as a direct cause of ED. Subgroup analysis studies from clinical databases can help us identify the specific effects of different factors on disease occurrence. Fourth, our study did not further explore how the pathophysiological changes caused by hypertension lead to ED. In the future, we will use mediated Mendelian analysis to further explore whether hypertension affects ED through different pathways such as circulating cytokines, sex hormone levels, plasma proteomics, etc. Finally, due to the lack of GWAS data on ED in Asian or African populations, only populations of European ancestry were included in this MR analysis, and our findings may not be applicable to other populations. Future researchers can further analyze the impact of hypertension on ED in different populations.

In conclusion, the results of this study suggest that genetically predicted hypertension increases the risk of ED, but we found no causal relationship between elevated SBP/DBP and ED. We speculate that the relationship between elevated BP and ED risk may be nonlinear. We found no evidence that the use of ACEIs, BBs, CCBs, and thiazide diuretic increased the risk of ED.

## Data Availability

The datasets presented in this study can be found in online repositories. The names of the repository/repositories and accession number(s) can be found in the article/**Supplementary Material**.
